# Environmental Factors Affecting Sleep Quality in Intensive Care Unit Patients in Southern Morocco: An Assessment Study

**DOI:** 10.3390/arm93030014

**Published:** 2025-06-06

**Authors:** Abdelmajid Lkoul, Keltouma Oumbarek, Youssef Bouchriti, Asmaa Jniene, Tarek Dendane

**Affiliations:** 1 Laboratory of Biostatistics, Clinical Research and Epidemiology, Faculty of Medicine and Pharmacy, Mohammed V University, Rabat 10010, Morocco; oumbarekeltouma@gmail.com (K.O.); dendanetarek@gmail.com (T.D.); 2 Geosciences, Environment and Geomatic Laboratory, Department of Earth Sciences, Faculty of Sciences, Ibn Zohr University, Agadir 80000, Morocco; youssef.bouchriti@gmail.com; 3 Laboratory of Physiology, Exercise Physiology and Autonomic Nervous System Team, Faculty of Medicine and Pharmacy, Mohammed V University, Rabat 10010, Morocco; a.jniene@um5r.ac.ma; 4 Department of Pulmonology, Ibn Sina University Hospital Center, Mohammed V University, Rabat 10010, Morocco

**Keywords:** intensive care unit, sleep questionnaire, sleep quality, sleep disorders

## Abstract

**Highlights:**

**What are the main findings?**

- Sleep deprivation is one of the most underestimated complications of the intensive care unit.
- Noise and light exposure were the most significant factors disturbing the patient’s Sleep.

**What is the implication of the main finding?**

- Improvements in pain management and minimization of environmental noise and staff disturbance could significantly improve the sleep quality of intensive care patients.

**Abstract:**

Introduction: Sleep disturbances are a common and often underestimated complication during intensive care unit (ICU) stays. These disturbances can significantly impact patients’ recovery and overall well-being. This study aimed to assess the sleep quality of ICU patients and investigate the environmental and clinical factors that affect sleep quality during their ICU stay. Methods: We conducted a six-month cross-sectional study involving patients who had stayed in the ICU for at least three nights and were oriented to time and place upon discharge. Sleep quality was assessed using the Arabic version of the Freedman Sleep Questionnaire. Both environmental factors (e.g., noise, light, and nursing interventions) and clinical variables (illness severity and pain) were examined. The differences across three time periods were analyzed using the Wilcoxon test and Spearman’s correlation. Multiple regression analysis identified the factors influencing sleep quality. Statistical analyses were performed using JAMOVI software (version 2.3.28). Results: The study enrolled 328 patients, with an average age of 49.74 ± 17.89 years. Of the participants, 75.3% were adults. The primary reasons for admission were circulatory distress (45.73%) and metabolic disorders (24.09%). Sleep quality was significantly lower in the ICU compared to patients’ sleep at home (Z = −14.870, *p* < 0.001). The EVA and APACHE II scores had a statistically significant effect on sleep quality (*p* < 0.001 and *p* = 0.015, respectively). In contrast, the Charlson and Quick SOFA scores did not show significant effects (*p* = 0.128 and *p* = 0.894). Environmental factors, including noise (*p* = 0.008), light (*p* = 0.009), and nursing interventions (*p* = 0.009), significantly impacted sleep quality. Conclusions: Patients in the ICU generally reported poor sleep quality. Our findings suggest that improving pain management, minimizing environmental noise, and reducing staff-related disturbances could significantly enhance sleep quality for patients in the intensive care unit (ICU).

## 1. Introduction

Sleep disturbances are a common but often under-recognized complication among critically ill patients admitted to the Intensive Care Unit (ICU), affecting over 61% of ICU patients globally [1]. These disturbances significantly affect patient recovery, contributing to an increased risk of infections, endocrine imbalances, impaired glucose tolerance, and heightened sympathetic nervous system activity [2,3,4,5]. Sleep is frequently fragmented in the ICU, with studies showing a decrease in total sleep time and a disruption in the natural sleep architecture. Specifically, ICU patients experience an increase in lighter sleep stages (N1 and N2), while restorative deep sleep (N3) and rapid eye movement (REM) sleep are either significantly reduced or completely absent [6].

Environmental factors, particularly noise and light, have been consistently identified as significant contributors to sleep disturbances in intensive care unit (ICU) settings [7,8,9]. However, it is essential to acknowledge that sleep disruption in the ICU is not solely attributable to these environmental factors. Routine diagnostic procedures, nursing interventions, and other aspects of ICU care can lead to frequent arousal and sleep fragmentation, further aggravating the problem [7,8,9,10]. Additionally, non-environmental factors, such as the patient’s underlying medical conditions (e.g., chronic obstructive pulmonary disease and myocardial infarction) and the treatments administered (e.g., sedatives and mechanical ventilation), have been identified as contributing to poor sleep quality [11,12,13,14].

Given the critical nature of ICU patients’ conditions, accurate and reliable sleep assessments are crucial for identifying these disruptions. While objective tools, such as polysomnography and actigraphy, are considered the gold standards for sleep assessment [15,16], these methods are not universally available, especially in resource-limited intensive care unit (ICU) settings. Moreover, their interpretation requires specialized expertise. In these contexts, subjective sleep assessment tools, such as questionnaires, are often employed. Despite the potential for bias inherent in these subjective measures, they offer a more feasible and cost-effective alternative for large-scale studies. The PADIS (Pain, Agitation, Delirium, Immobility, and Sleep) guidelines, developed by the Society of Critical Care Medicine, propose a multi-component approach to mitigate sleep disturbances in ICU patients. These guidelines aim to improve sleep quality by addressing factors related to pain, agitation, and immobility, thereby enhancing patient outcomes [17].

Despite the extensive documentation of the adverse effects of sleep deprivation on ICU patients globally, there is a notable lack of research examining the factors influencing sleep quality in Moroccan ICUs. This gap is significant given that the ICU environment in Morocco may present unique challenges due to regional healthcare practices, cultural differences, and environmental factors. Previous studies have primarily focused on Western or high-income country settings, and their findings may not directly apply to the Moroccan context.

This study aims to assess the sleep quality of ICU patients in southern Morocco and analyze the environmental (noise, light, and nursing interventions) and clinical factors (pain and illness severity) influencing their sleep during ICU admission. We will use a combination of subjective (Freedman Sleep Questionnaire) and objective (clinical indicators, such as APACHE II and pain scores) tools to assess the impact of these factors. The study also aims to fill the gap in the regional literature by examining the specific environmental and clinical factors that influence sleep quality in Moroccan intensive care units (ICUs). The ultimate goal is to provide actionable insights that inform targeted interventions to improve the sleep quality and overall well-being of critically ill patients in Moroccan intensive care units (ICUs).

## 2. Methods

This cross-sectional study was conducted among adult patients admitted to the Intensive Care Unit (ICU) at three public hospitals in the southern region of Morocco. Ethical approval was granted by the Committee for Biomedical Research Ethics (CERB) of the Faculty of Medicine and Pharmacy of Rabat (approval number: File No. 85/24). Furthermore, data collection was approved by the regional Department of Health and Social Protection in the Souss-Massa region. Before participating, all patients received a comprehensive explanation of the study’s aims, procedures, and potential risks and provided their written informed consent.

The study employed a convenience sampling method, where patients were selected based on their availability and willingness to participate in the study. Although this approach is practical, it may introduce a potential selection bias. This issue will be addressed in the limitations section of the survey. Between May and December 2024, 494 patients were admitted to the intensive care units (ICUs) of the selected hospitals. Of these, 328 patients met the inclusion criteria and agreed to participate, completing all aspects of the study. Based on the existing literature, we assumed a prevalence of 61% for sleep disturbances in ICU patients for the sample size calculation. With a 5% margin of error and a 95% confidence interval, the calculated sample size required was 329 participants. Since the study included 328 participants, the sample size was sufficient for the statistical analyses. Selection biases were minimized using a more diverse sample in terms of age, pathologies, and severity of illness; the comparison between the characteristics of participants and non-participants revealed no significant differences, and the increase in the number of participants, which exceeded 94% of the targeted population in the study.

Data were collected at three distinct time points during the patient’s ICU stay: at the beginning, the middle, and the end of their hospitalization. The initial data collection occurred upon patient admission to the ICU, mid-stay data were gathered approximately two days after admission, and final data were collected upon discharge or transfer. The methodology for data collection at these three time points is outlined in the study protocol to ensure consistency in data capture and reduce potential biases associated with varying assessment timelines.

The following sociodemographic variables were recorded: age, gender, marital status, geographic origin (urban or rural), profession, and education level. Additionally, medical data, including body mass index (BMI), immune status, and reason for admission to the ICU, were documented. Hospitalization-related factors were also recorded, including the length of stay, type of intensive care unit (ICU) care, health insurance coverage, and level of regular physical activity before admission. These data were collected through structured questionnaires, supplemented by a review of the patient’s medical records, ensuring accurate and reliable information.

To assess sleep quality, we utilized the Arabic version of the Freedman Sleep Questionnaire, which had been previously validated in our research, yielding a Cronbach’s alpha of 0.816 and an intraclass correlation coefficient (ICC) of 0.85. This tool allowed for a thorough evaluation of both subjective sleep disturbances and the factors influencing sleep quality in critically ill patients. In addition to sleep quality, we assessed various environmental factors within the ICU, such as noise levels, that contribute significantly to sleep disturbances. High sound levels in the ICU were defined as sounds perceived as disruptive and exceeding patients’ comfort levels. These levels were determined based on subjective observations and patient feedback. The threshold for high sound levels was set at any sound intensity exceeding 50 decibels, which aligns with the existing literature on environmental noise in intensive care unit (ICU) settings. This threshold was chosen because exposure to prolonged noise above this level has been linked to elevated stress hormones, impaired sleep architecture, and delayed recovery. Data on noise levels were recorded using an objective sound measurement tool, such as a decibel meter, during the data collection process. Several standardized clinical scores were also recorded for each participant to assess the severity of their condition. These included the Charlson Comorbidity Index, which categorizes mortality risk into three levels (0–1: low, 2–3: moderate, and ≥4: high) based on the presence of comorbidities. The quick SOFA score is used to assess sepsis risk and is categorized into three levels: 0 (low), 1–2 (moderate), and 3 (high). The EVA-pain score, which assesses pain severity, is categorized into three levels: 0–3 (low), 4–7 (moderate), and 8–10 (high), and the APACHE II score is categorized into four levels of mortality risk (0–4: low, 5–9: moderate, 10–19: high, and >19: very high) based on the severity of the underlying condition.

The data were examined using suitable statistical methods, considering their distribution. The Kolmogorov–Smirnov test was initially used to assess the normality of the sleep quality data. The Wilcoxon signed-rank test was used to evaluate the difference in sleep quality between the ICU and home settings, as the data were non-normally distributed. Additionally, the evolution of sleep quality throughout the ICU stay was analyzed using the Wilcoxon test to detect any significant changes over time. Multiple linear regression analyses were conducted to examine the relationships between sleep quality and its influencing factors. The dependent variable was sleep quality, and the independent variables included clinical scores (Charlson, SOFA, EVA, and APACHE II) and various environmental factors, such as noise levels, light intensity, nursing interventions, and the use of medical equipment within the intensive care unit (ICU). Standardized and unstandardized regression coefficients, 95% confidence intervals, *p*-values, tolerance values, and the variance inflation factor (VIF) were calculated to assess the significance and strength of each predictor while controlling multicollinearity among the independent variables.

## 3. Results

This study examined the sociodemographic characteristics, medical conditions, and hospital stays of 328 patients admitted to the intensive care unit (ICU). The results presented in this paper focus on the sleep quality of these patients, comparing their sleep in the ICU with their sleep at home and analyzing factors that influence their sleep quality in the ICU. Statistical analyses, including non-parametric tests (Wilcoxon) and multiple linear regressions, were employed to investigate the relationships between sleep quality and various variables, such as the EVA, APACHE II, Charlson, and SOFA scores, as well as service activities and noise levels in the ICU.

### 3.1. Sociodemographic Characteristics of Patients Admitted to Intensive Care

Table 1 presents the distribution of sociodemographic characteristics, medical conditions, and hospital stays for a sample of 328 individuals. The average age was 49.74 ± 17.89 years (range: 18–93 years), with a median age of 50 years. A total of 75.3% of the participants were adults (18–65 years), and 24.7% were 65 and older. The average BMI was 23.06 ± 4.60 kg/m^2^ (15 to 41 kg/m^2^), with a median of 22 kg/m^2^. The BMI distribution was 45.73% with normal weight (18.5–24.9 kg/m^2^), 25% with overweight (25–29.9 kg/m^2^), 8.54% with obesity (30–34.9 kg/m^2^), 1.22% with morbid obesity (>35 kg/m^2^), and 19.51% with underweight (<18.4 kg/m^2^). The average length of stay was 5.20 ± 2.44 days (2 to 16 days), with a median of 4 days.

Regarding the other variables presented in Table 1, there was almost an equal distribution between men (49.09%) and women (50.91%). Most participants were married (75%), followed by singles (14.94%). The origin of the patients was nearly equivalent between rural areas (52.44%) and urban areas (47.56%). A total of 40.85% had no formal education, 29.88% had primary education, 23.78% had secondary education, and 5.49% had university-level education. A total of 86.59% of participants did not engage in regular physical activity, and 53.96% were immunocompetent. The primary admission reasons were circulatory distress (45.73%), followed by metabolic disorders (24.09%), respiratory distress (25%), and other reasons (5.18%). Finally, most ICU admissions were medical (71.34%).

The 95% confidence intervals for the mean age, BMI, and length of stay were [47.80; 51.69] years, [22.56; 23.56] kg/m^2^, and [4.94; 5.47] days, respectively.

### 3.2. Comparative Analysis of Sleep Quality: Intensive Care Unit (ICU) and Home Environment

Table 2 compares sleep quality in the ICU and at home. This comparison reveals a significant difference. Patients reported significantly poorer sleep quality in the ICU (mean score of 4.02 ± 2.04, with a range of 1 to 10) compared to at home (mean score of 6.55 ± 1.80, with a range of 2 to 10). The non-normal distribution of the scores, confirmed by the Kolmogorov–Smirnov tests (*p* < 0.001 for both), led to the use of the Wilcoxon rank-sum test. This test strongly indicates a difference in sleep quality between the two environments (Z = −14.870, *p* < 0.001), with significantly lower ranks for sleep quality in the ICU. This conclusion is further supported by individual rankings: 297 patients reported worse sleep in the ICU, only 4 reported better sleep, and 27 noticed no difference.

### 3.3. Analysis of Sleep Quality and Daytime Sleepiness Throughout the ICU Stay (Start, Middle, End)

Sleep quality was assessed at various time points and compared to that in the ICU (Table 3). In the intensive care unit, the average sleep quality was 4.02 ± 2.04 on a scale of 1 to 10. At the beginning of the study, the average was 4.44 ± 2.03, which improved to 4.60 ± 2.04 at the midpoint and further improved to 4.81 ± 2.16 by the end of the study. The average sleep quality over the three days was compared to the sleep quality at three time points during the ICU stay: the beginning, middle, and end. The results indicate that the worst sleep quality was observed at the beginning, with notable improvement by the end of the ICU stay.

The Wilcoxon test compared sleep quality on these nights to overall sleep quality during the ICU stay. The comparison between initial sleep quality and sleep quality in the ICU revealed 68 negative ranks, 106 positive ranks, and a Z value of −4.859 (*p* < 0.001). The comparison between sleep quality at midnight and overall sleep quality in the ICU showed 49 negative ranks, 115 positive ranks, and a Z value of −6.804 (*p* < 0.001). Finally, the comparison between sleep quality at the end of the stay and sleep quality in the ICU indicated 15 negative ranks, 124 positive ranks, and a Z value of −9.093 (*p* < 0.001). These results, based on the negative ranks, indicate a statistically significant improvement in sleep quality from the beginning to the end of the study compared to the ICU’s baseline.

For the comparison between daytime sleepiness on the first day and overall ICU daytime sleepiness, a negative rank (indicating lower sleepiness on the first day) accounted for 112 observations. In comparison, positive ranks (higher sleepiness on the “First day”) included 32 observations. Tied ranks were observed in 184 cases. The two-tailed significance value was <0.001, indicating significantly higher daytime sleepiness in the ICU compared to the “First day”.

Regarding the comparison between the “Middle day” and overall ICU daytime sleepiness, the negative rank (90 observations) outnumbered the positive rank (38 observations). The *p*-value of <0.001 confirms significantly greater daytime sleepiness in the ICU compared to the “Middle” day.

Conversely, for the comparison between the “last day” and overall ICU daytime sleepiness, negative (38 observations) and positive (43 observations) ranks were nearly balanced. The *p*-value of 0.330 indicates no statistically significant difference between these two days, suggesting a similar level of daytime sleepiness at the end and during the ICU stay.

### 3.4. Correlation Between Sleep Quality and Clinical Scores in ICU Patients

Table 4 presents a multiple linear regression to predict sleep quality in the ICU based on the APACHE II, EVA, Charlson, and SOFA scores. The model explains 10.4% of the variance in sleep quality (R^2^ = 0.104, adjusted R^2^ = 0.093). The standard error of the estimate is 1.946. The Durbin–Watson statistic (2.051) indicates no autocorrelation of residuals, validating one of the assumptions of linear regression. The EVA and APACHE II scores have a statistically significant effect on sleep quality (*p* < 0.001 and *p* = 0.015, respectively). In contrast, the Charlson and Quick Sofa scores are unimportant (*p* = 0.128 and *p* = 0.894). The absence of multicollinearity between the predictors strengthens the validity of the model.

Unstandardized coefficients (B) indicate the effect of each predictor on sleep quality in raw units. A negative coefficient indicates a negative association: an increase in the predictor’s score is associated with a decrease in sleep quality. For example, for each increase of one unit in the EVA score, sleep quality decreases by 0.145. Standardized coefficients (Beta) allow for the comparison of the relative importance of different predictors. The EVA score has the most significant effect (−0.228), followed by the APACHE II score (−0.157). The 95% confidence intervals for the B coefficients indicate the range of plausible values for each predictor’s real effect on the population. Collinearity statistics (tolerance and VIF) suggest no multicollinearity issues between the predictors. All tolerance values are above 0.3 (and even above 0.6), and all VIF values are below 4 (and even below 2). This result means the predictors are not strongly correlated with each other, further validating the model.

### 3.5. Association Between Patients’ Sleep Quality and ICU Care Activities and Noises

Table 5 presents a multiple linear regression to understand the factors influencing sleep quality in the ICU. The model tested utilizes various environmental and healthcare-related variables as predictors. Overall, the model explains about 19.1% of the variability in sleep quality (R^2^ = 0.191). This result is confirmed by a significant ANOVA test (*p* < 0.001), indicating that the model is generally relevant. The adjusted R^2^, at 0.152, is lower, suggesting that some variables may be redundant or less important, such as vital signs and Ventilator alarm, which were removed from the model due to their high correlation with oxygen finger probe (r = 0.864, *p* < 0.001) and ventilator sound (r = 0.992, *p* < 0.001), respectively, and their very high multicollinearity, with a VIF of 5.132 and 66.543.

An analysis of the individual coefficients reveals that several factors have a statistically significant impact on sleep quality. Noise (*p* = 0.008), light (*p* = 0.009), IV pump (*p* =0.038), nursing intervention (*p* = 0.009), and heart monitor alarm (*p* < 0.001) significantly influence sleep quality. Although included in the model, other variables do not show a statistically significant effect at a 5% threshold. These include diagnostic testing (*p* = 0.818), medication (*p* = 0.877), blood sampling (*p* = 0.137), ventilator noise (*p* = 0.653), oxygen finger probe (*p* = 0.200), conversations (*p* = 0.178), suction noise (*p* = 0.232), and nebulization noise (*p* = 0.197). For the significant variables, the coefficient sign indicates the direction of the relationship: a negative coefficient suggests that an increase in the predictive variable is associated with a decrease in sleep quality.

The collinearity statistics (tolerance and VIF) indicate an absence of multicollinearity between the predictors.

## 4. Discussion

This study aimed to identify sleep disturbances associated with sitting in an environment among ICU patients using a self-administered questionnaire. This study was conducted in three public hospitals located in southern Morocco. The multifactorial nature of sleep disruption in the ICU, combined with the vulnerability of critically ill patients, complicates the identification of factors that impact sleep quality. It is clear, however, that these patients experienced a significant deterioration in sleep quality during their ICU stay compared to sleep at home. Our findings align with those of Freedman and the other existing literature. Prior studies have shown that ICU patients experience notably poorer sleep quality compared to at home [4,6,11,18]. While we did not find statistically significant differences in sleep during the ICU stay, as reported by Al Mutair et al. [2], we documented a substantial reduction in patient drowsiness by the end of the ICU admission. Furthermore, most patients experienced daytime sleepiness, indicative of inadequate nighttime rest. In contrast to many previous studies, we expanded our investigation to include a broader range of factors that may influence sleep quality. Beyond the commonly studied environmental factors, such as those described by Freedman et al. [19], our analysis also considered non-environmental factors, including the severity of illness, ICU length of stay, and pain levels. We deemed it essential to examine both clinical and environmental factors to better understand the root causes of sleep disruption in the ICU. Numerous reports highlight the importance of sleep disorders as a significant issue for ICU patients, and our study is the first of its kind in Morocco to address this concern. In our multivariate analysis, both environmental and non-environmental factors were found to impact the quality of sleep during ICU stays. This study did not identify any significant relationships between patients’ demographic characteristics and their perceived quality of sleep. The literature presents mixed findings regarding the influence of demographic factors on sleep quality. For instance, the results of this study align with those of Younis, who conducted research in Jordan and similarly reported no significant correlations between demographic data and sleep quality [13]. Additionally, Boyko et al. found no significant correlation between age, gender, or sedation regimen and abnormal sleep patterns, as evaluated by polysomnography [20].

Regarding clinical factors, our results did not reveal a significant impact of postoperative status on sleep quality. However, previous research indicates that deep sleep is often reduced or absent immediately following surgery and anesthesia [21,22]. This discrepancy may be attributed to the limited proportion of postoperative cases in our sample, which accounted for only 28%. Also, the study was conducted in multidisciplinary intensive care units, a context that likely hindered the precise identification of this factor’s influence. Interestingly, no significant correlations were observed between sleep quality and variables such as infection severity (as measured by the SOFA score) and patients’ comorbidities (as assessed by the Charlson score). This finding may be explained by the fact that most patients in our study were not in sepsis or experiencing severe organ dysfunction. Since the SOFA score assesses multi-organ failure, its impact on sleep quality was limited. Additionally, the use of antibiotics and anti-inflammatory medications could have influenced the results. However, the illness severity (the APACHE II score) in our study has a statistically significant effect on sleep quality (*p* < 0.015), in contrast to several studies that showed no significant correlation between APACHE II and sleep quality [23,24]. This discrepancy could be attributed to the specific context of the ICUs in which the previous studies were conducted, as their focus was limited to neurology patients in one case and pulmonology patients in the other. In contrast, our study was conducted in a setting where patients exhibited a diverse range of pathologies with varying degrees of severity. This heterogeneity in patient profiles may have influenced the observed outcomes, highlighting the importance of considering variability in clinical presentations when interpreting results.

The pain intensity (the EVA score) appeared to have a statistically significant effect on sleep quality (*p* < 0.001), aligning with findings in previous studies [2,13,14,25,26]. This finding can be attributed to inadequate management and assessment of pain in the ICU. Pain management practices predominantly involve the administration of paracetamol and anti-inflammatory medications. While these may provide some relief, they are often insufficient in addressing severe nociceptive pain or neuropathic pain, which requires a more comprehensive approach. This inadequate pain control can also negatively impact the quality of sleep, as unrelieved pain often disrupts sleep patterns, leading to poor recovery and overall decreased patient prognosis.

Furthermore, an improvement in sleep quality was noted with increased ICU stay duration. This finding contrasts with previous research, including studies by Al Mautair et al. [2], which indicated no significant differences in sleep between the first and last nights in the ICU, and as reported in the study conducted by Freedman et al. [27] on 24 ICU patients using polysomnography. These results suggest that while extended hospitalization may partially mitigate sleep disturbances, the environmental disruptions inherent to the ICU remain significant and cannot fully substitute the restorative benefits of sleep in a familiar setting.

Previous authors have estimated that ICU environmental factors contribute to patient arousal and awakenings in this setting. Machine noise-related disruptions to sleep quality in the ICU have been well-documented [4,7,8,9,11,13,14,23,28]. In our study, the most disruptive noises were those from heart monitor alarms and IV pumps. One possible solution to mitigate this problem is to reduce the volume of monitor sounds and alarms, emphasizing the need for a prompt response to alarms to minimize disturbances.

Our findings confirm that noise in the ICU is a significant factor affecting sleep quality, aligning with previous studies’ observations. Noise has been shown to induce arousal without full awakening, contributing to sleep fragmentation and a subsequent decline in sleep quality. The average acceptable noise level in a hospital, as recommended by the World Health Organization, should not exceed 40 dB during nighttime hours [28]. In addition to environmental noise, patients highlighted nursing activities and medical interventions, particularly the measurement of vital signs, as significant disruptors of their sleep. Specifically, blood pressure cuffs were frequently identified as a primary source of disturbance. This observation is consistent with other studies that emphasize the impact of routine nursing care on sleep disruption [9,11,13,26,28]. These results highlight the importance of minimizing environmental noise and optimizing care routines to mitigate their adverse effects on patient sleep quality in the intensive care unit (ICU).

Light exposure was the second most significant factor, likely due to the design of many ICU environments that often lack individualized patient areas with adequate shading or control over light exposure. This environmental shortcoming can disrupt circadian rhythms and adversely affect sleep quality. In many ICUs, lights remain on throughout the unit, further exacerbating this disruption. These findings align with Pamuk et al.’s research, which highlighted the detrimental impact of light exposure on patients’ nighttime rest, emphasizing the need for improved lighting strategies to support circadian alignment and enhance patient recovery [10].

Although numerous studies have been conducted on sleep quality in the ICU, this remains a complex and unresolved issue due to its multifactorial nature. Clinical guidelines for sedo-analgesia management in critically ill adults strongly recommend non-pharmacological measures, such as reducing nighttime noise and adjusting light exposure, to improve sleep quality by reducing sleep fragmentation [5,16,17,24]. Adopting these guidelines is essential, as sleep disturbances extend beyond the ICU.

The weak explanatory power of the models (R^2^ = 0.104 for clinical variables and R^2^ = 0.191 for environmental factors) may stem from several factors. First, the clinical and environmental variables included in the models may not fully capture the complexity and multifactorial nature of sleep disturbances in ICU patients. Sleep quality in critically ill patients is influenced by a wide range of interconnected factors, including the severity of the illness, medication side effects, psychological stress, and the overall ICU environment, which may not have been fully accounted for in this study. Second, the relatively small sample size of 328 participants, although adequate for statistical power, may still limit the models’ ability to detect all significant relationships between the variables. Small sample sizes can lead to overfitting or underfitting of the models, resulting in weaker explanatory power. Additionally, the data collection process may have introduced measurement errors or omitted important variables, particularly those related to environmental factors. For instance, noise levels in the ICU could vary over time, and only certain aspects were measured, potentially oversimplifying the ecological factors that contribute to sleep disturbances.

The recent literature highlights several advancements in improving sleep quality for ICU patients, building on prior practices. Notably, the use of non-invasive sleep monitoring technologies allows for accurate tracking without disturbing the patient’s environment. Additionally, personalized approaches to noise and light management, tailored to individual needs, enhance the sleep environment by respecting the circadian rhythm. Current sedation strategies focus on lighter sedation combined with non-pharmacological interventions, such as relaxation techniques, to preserve natural sleep cycles while managing pain and anxiety. Moreover, the adoption of sleep-centered care protocols, which aim to optimize the sleep environment, marks a shift from more generalized approaches. Ultimately, raising awareness and educating healthcare staff on the importance of sleep in recovery ensures that these strategies are consistently implemented, leading to improved patient outcomes.

Further research is essential to better understand and address sleep quality among ICU patients. Several critical aspects remain underexplored, such as the effects of depression, anxiety, invasive ventilation, and ventilatory modes on sleep, as well as the impact of inotropic or cardiovascular-active drugs on sleep deprivation. Moreover, there is a clear need for additional multicenter studies to evaluate targeted interventions and strategies to promote sleep and minimize disruptions to sleep–wake patterns in ICU settings. Such studies should consider key variables, including disease severity, patient age, medication regimens, and length of ICU stay, to develop more comprehensive and practical approaches to improving sleep outcomes for critically ill patients.

## 5. Conclusions

The results of this study highlight the significant impact of ICU environmental factors on patients’ perceived sleep quality, with noise, light exposure, and nursing activities being key disruptors of sleep. To improve sleep quality and support recovery, it is essential to implement strategies such as noise reduction, light optimization, and appropriate sedation management. Reducing noise from alarms and staff conversations, using soundproofing, and adjusting lighting to support the circadian rhythm can create a more restful environment. Additionally, managing sedation levels to avoid over-sedation, combined with non-pharmacological interventions like relaxation techniques, helps preserve natural sleep cycles. Raising awareness among healthcare staff about the importance of sleep and fostering a culture that prioritizes sleep can ensure these practices are consistently applied. By addressing these environmental factors, ICUs can significantly enhance patient well-being, reduce complications, and optimize recovery outcomes.

### Limitations

This study has several limitations that should be acknowledged. Firstly, this study used a cross-sectional design, which limits the ability to establish causal relationships. Although associations between factors and sleep quality were observed, causality cannot be confirmed. Furthermore, selection biases may have affected the results, as only patients present at a specific time were included, potentially not representing the entire ICU population. Secondly, sleep quality and its influencing factors were assessed subjectively using the validated Arabic version of the Freedman Questionnaire. While this tool is useful, its reliance on personal assessments introduces potential biases, making it less reliable than objective methods, such as polysomnography or actigraphy, which provide more accurate and precise data. However, this limitation had a minimal impact on our results, as previous research by Freedman has indicated that recall bias is not a significant concern over the relatively short recall periods typically associated with ICU stays. Another limitation lies in our methodology, as we did not incorporate objective tools, such as actigraphy, a cost-effective and low-labor option for monitoring sleep–wake cycles in intensive care unit (ICU) settings. Additionally, recruitment and selection biases may have occurred, which could limit the generalizability of our findings to the broader ICU population. Finally, this study focused on assessing quantitative factors that disrupt sleep in the ICU. Psychological factors such as stress, depression, fear, and mood, which are known to influence sleep quality significantly, were not evaluated. Addressing these limitations in future studies could provide a more comprehensive understanding of sleep disturbances in ICU patients.

## Figures and Tables

**Table 1:** Sociodemographic Characteristics of Patients Admitted to Intensive Care (n = 328).

Variables/Modalities	n (%) ^a^	95% LCL–LCU ^b^
Age		
18–65	247 (75.30)	70.43–29.57
≥65	81 (24.70)	20.26–29.57
BMI		
<18.4	64 (19.51)	15.50–24.06
18.5–24.9	150 (45.73)	40.40–51.14
25–29.9	82 (25.00)	20.55–29.89
30–34.9	28 (8.54)	5.87–11.93
≥35	4 (1.22)	0.41–2.87
Gender		
Male	161 (49.09)	43.70–54.48
Female	167 (50.91)	45.52–56.30
Marital status		
Single	49 (14.94)	11.40–19.10
Divorced	11 (3.35)	1.79–5.73
Married	246 (75.00)	70.11–79.45
Widowed	22 (6.71)	4.37–9.80
Origin		
Rural	172 (52.44)	47.03–57.80
Urban	156 (47.56)	42.20–52.97
Level of education		
None	134 (40.85)	35.63–46.23
Primary	98 (29.88)	25.12–34.99
Secondary	78 (23.78)	19.42–28.60
University	18 (5.49)	3.40–8.35
Physical activity		
No	284 (86.59)	82.58–89.95
Yes	44 (13.41)	10.05–17.42
Immunization status		
Immunocompetent	177 (53.96)	48.55–59.30
Immunocompromised	151 (46.04)	40.70–51.45
Reason for admission		
Other	17 (5.18)	3.17–7.99
Metabolic disorder	79 (24.09)	19.70–28.93
Circulatory distress	150 (45.73)	40.40–51.14
Respiratory distress	82 (25.00)	20.55–29.89
Type of intensive care		
Surgical	38 (11.59)	8.46–15.38
Medical	234 (71.34)	66.28–76.03
Postoperative	56 (17.07)	13.30–21.43

^a^: n: sample size, %: percentage; ^b^: LCL: lower confidence limit at 95%, UCL: upper confidence limit at 95%.

**Table 2:** Wilcoxon test on sleep quality between ICU and home in patients admitted to the ICU n = 328.

Variables	M ± SD	Min–Max	*p* (K-S) ^a^	Positive Rankings	Negative Rankings	Ranks Ex-Aequo	Z ^b^	*p* ^c^
Sleep quality in the ICU	4.02 ± 2.04	1–10	<0.001	-	-	-	-	-
Sleep quality at home	6.55 ± 1.80	2–10	<0.001	-	-	-	-	-
Sleep quality ICU– sleep quality at home	-	-	-	297 ^d^	4 ^e^	27 ^f^	−14.870	<0.001

^a^: Kolmogorov–Smirnov normality test *p*-value. ^b^: Z test based on positive rankings. ^c^: Wilcoxon’s two-sided asymptotic significance of the statistical rank test. ^d^: Sleep quality ICU < sleep quality at home; ^e^: Sleep quality ICU > sleep quality at home; ^f^: Sleep quality ICU = sleep quality at home.

**Table 3:** The analysis of sleep quality and daytime sleepiness throughout the ICU stay (start, middle, end).

Variables	M ± SD	Min–Max	*p* (K-S) ^a^	Negative Rankings (Average Rank)	Positive Rankings (Average Rank)	Ranks Ex Aequo	Z ^b^	*p* ^c^
Sleep quality ICU	4.02 ± 2.04	1–10	<0.001	-	-	-	-	-
Sleep Quality First	4.44 ± 2.03	1–10	<0.001	68 (65.40)	106 (101.68)	154	−4.859	<0.001
Sleep Quality Middle	4.60 ± 2.04	1–10	<0.001	49 (55.00)	115 (94.22)	164	−6.804	<0.001
Sleep Quality End	4.81 ± 2.16	1–10	<0.001	15 (39.27)	124 (73.72)	189	−9.093	<0.001
Day Time Sleepiness ICU	6.16 ± 2.06	2–10	<0.001	-				
Day Time Sleepiness First	5.48 ± 2.21	1–10	<0.001	112 (77.15)	32 (56.23)	184	−6.933	<0.001
Day Time Sleepiness Middle	5.76 ± 2.02	1–10	<0.001	90 (71.94)	38 (46.88)	200	−5.685	<0.001
Day Time Sleepiness End	6.08 ± 2.09	2–10	<0.001	38 (49.05)	43 (33.88)	247	−0.974	0.330

^a^: Kolmogorov–Smirnov normality test *p*-value. ^b^: Z test based on positive rankings. ^c^: Wilcoxon’s two-sided asymptotic significance of the statistical rank test.

**Table 4:** Multiple linear regression of ICU sleep quality based on EVA, Charlson, Sofa, and Apache II Scores n = 328.

Model	β ^a^	Standard Error	*p* ^b^	95% CI (β) ^c^	Tolerance ^d^	VIF ^e^
(Constante)	5.241	0.236	<0.001	4.777–5.704	-	-
EVA Score	−0.145	0.034	<0.001	−0.212–−0.078	0.975	1.026
Charlson Score	−0.131	0.086	0.128	−0.301–0.038	0.880	1.136
SOFA Score	0.021	0.154	0.894	−0.283–0.324	0.746	1.340
APACHE II Score	−0.055	0.023	0.015	−0.100–−0.011	0.670	1.492

^a^: Unstandardized coefficients; ^b^: statistical significance of the Student’s *t*-test; ^c^: 95% confidence interval of the unstandardized coefficients β; ^d^: collinearity test using tolerance measurement; ^e^: collinearity test using variance inflation factor (VIF).

**Table 5:** Multiple linear regression of ICU sleep quality based on care activities and noises.

Model	β ^a^	Standard Error	*p* ^b^	95% CI (β) ^c^	Tolerance ^d^	VIF ^e^
(Constante)	5.238	0.534	<0.001	4.187–6.290		
Noise	−0.200	0.075	0.008	−0.347–−0.052	0.762	1.312
Light	0.214	0.082	0.009	0.053–0.375	0.333	3.004
Nursing intervention	0.558	0.213	0.009	0.138–0.978	0.887	1.127
Diagnostic testing	−0.018	0.078	0.818	−0.171–0.135	0.337	2.964
Blood samples	−0.096	0.065	0.137	−0.223–0.031	0.488	2.047
Medication	−0.010	0.063	0.877	−0.134–0.114	0.593	1.685
Heart monitor alarm	−0.448	0.116	<0.001	−0.676–−0.220	0.303	3.299
Ventilator sound	−0.032	0.071	0.653	−0.171–0.107	0.490	2.040
Oxygen finger probe	0.085	0.066	0.200	−0.045–0.214	0.478	2.091
Talking	−0.098	0.073	0.178	−0.242–0.045	0.347	2.884
IV pump	−0.053	0.060	0.038	−0.171–0.066	0.479	2.087
Suctioning	0.144	0.120	0.232	−0.093–0.381	0.288	3.473
Nebulizer	0.138	0.107	0.197	−0.072–0.348	0.330	3.034
Conversations	−0.117	0.088	0.184	−0.290–0.056	0.304	3.294
Personal telephone	−0.107	0.188	0.569	−0.477–0.262	0.943	1.060

^a^: Unstandardized coefficients; ^b^: statistical significance of the Student’s *t*-test; ^c^: 95% confidence interval of the unstandardized coefficients β; ^d^: collinearity test using tolerance measurement; ^e^: collinearity test using variance inflation factor (VIF).

## Data Availability

The dataset is available upon request from the authors.
